# Graduate Study on the Continent—Austria

**Published:** 1909-01-16

**Authors:** 


### GRADUATE STUDY ON THE CONTINENT—AUSTRIA.

### VI.—GRAZ.

To English-speaking students this centre is probably totally unknown. Vienna absorbs so much attention that the claims of the smaller, and in many respects better, centres are quite overlooked. This is a lamentable fact when one considers that, as in Germany, the so-called provincial centres offer attractions which differ from those which are to be found in the capital. Nor must one lose sight of the fact that the large centres in Germany are exceptional when compared with the smaller ones in so far that the former offer a much more vast and valuable amount of clinical material; while at the latter, owing to the many centres and the large hospital accommodation, the amount of such material is limited. In Austria this is by no means the case. There are practically only five centres, and none of them is much inferior, as regards quality and quantity of clinical material available, to the capital. What is more, owing to the concentration of such material in such centres as Graz and Prague, these centres are thoroughly equal to Vienna from the post-graduate's point of view, in one respect at least.

As a centre Graz offers many attractions to the graduate, and those who have once enjoyed its hospitality will carry away with them pleasant recollections of their stay and the firm determination to visit it again. The city itself is not large, although it has a population of more than 150,000. It is an old town, full of interest to the ordinary tourist, and no less worthy the study of the medical graduate who delights in the contemplation of historical relics. From here as a base, easy excursions can be made into the delightful region of the Styrian Alps, where the climber can avail himself of the numerous facilities which the distinct branch of the Austrian Alpine Club has put at his disposal. So, too, the sportsman will find Graz a good centre. There are plenty of opportunities for fishing and shooting, both well preserved, but easily within reach of the man who can afford to devote a few pounds to the enjoyment of his pastime. Living at Graz is, on the whole, cheap, though room and house rents are relatively high when compared with the small German centres. The average price for a room in the city itself is about 26 kronen monthly, without attendance or breakfast. We ourselves, during our months of graduate study at Graz—a period to which we look back with more pleasure than to our study time at any other Continental centre—obtained lodgings at the comparatively cheap rent of 32 kronen per month. This was for a large room, with electric light and separate entrance, in the vicinity of the hospitals and city park, and with a fine balcony which overlooked the river Mur and commanded a grand view over the north-western mountain ranges. Breakfast averaged 60 heller, and lunch and dinner were easily obtainable at any of the restaurants, the food being everywhere good and the prices relatively cheap. There are several excellent hotels, all of them within easy reach of the clinics and railway station, and for a long stay, when the graduate brings his family with him, the numerous *pensions* can be recommended. In addition, flats and villas may be hired for the whole year, or for the winter or summer term. Thus a villa with four rooms is rented at an average price of 280 kronen for the season. The graduate is strongly advised to write to the Secretary of the Landes-Verband für Fremdenverkehr Speditionsfirma Franz Kloiber, Neutorgasse 42, Graz, for a copy of the year-book issued by the Society. This gives all information and will be found a very useful and valuable handbook. It is supplied free on application. We may here remark that Steiermark, of which Graz is the capital, is too little known to English tourists. It is one of the finest Austrian provinces, which will well repay an extended visit. Graz itself lies at an elevation of more than a thousand feet above the level of the Adriatic, and possesses an equable climate which should make it, in summer especially, a fine health resort. In winter the cold is considerable, but not excessive, and severe frosts are of very short duration.

We have devoted somewhat more space than usual to the introduction because in our opinion Graz is a centre that deserves the sympathetic attention of every graduate student who comes to Austria, and nothing is more astonishing than the fact that hitherto it has been the most neglected of Austrian centres so far as the English-speaking graduate is concerned. Just as the English tourist rarely comes to

Steiermark, notwithstanding its many attractions as a tourist centre, so the English medical graduate never comes to Graz, notwithstanding the fact that it should become one of the finest of post-graduate centres. The reason for this neglect is probably the fact that the attractions of town and district are not known in England or America. To Continental tourists and Continental practitioners they are thoroughly known. The fame of Graz as a medical centre is, however, not international. In Austria, Germany, Roumania, Servia, and even in Turkey and Russia, it is recognised that Graz is a centre of importance. Its medical faculty includes men who, like Professor von Hacker, have won a reputation as experts in their special branch and as clinical teachers. This appreciation of the good work that is done at Graz is proved by the fact that many German and Austrian Universities have recruited the best members of their staffs from the Styrian hospitals. Thus, to mention only a few names, Professor Kraus, of Berlin; Professor Pfaundler, of Munich; Professor Wölfler, of Prague; and Professor Roshorn, of Vienna—all names well known outside the limits of their various centres—were Professors at Graz, who made their reputation there before they were called to, and accepted, a chair at a larger centre. We lay stress on these facts because we are convinced that an acquaintance with the hospitals and the facilities at Graz will rapidly win for it the same appreciation from the English-speaking graduate which he now gives to better-known centres. At present the facilities for post-graduate study here are not all that they might be, and the centre holds out the best attractions to the pure free-lance. When Graz becomes better known, however, all this will be changed. The medical faculty and the staffs of the various professors fully recognise the advantages that they possess, and are thoroughly willing to cater for the graduate. "Let them come and we will provide work for them" is the invariable answer one gets when one sounds a member of the staff on the question of post-graduate courses. "At present, as you see, Herr Collega, they all flock to Vienna."

What are these advantages? We have already pointed out some of the attractions which Graz possesses as a centre for sojourn and recuperation. Clinically it offers equal attractions. There is a large Allgemeines Krankenkaus in the Paulusthor street, an old building after the style of the Vienna General Hospital, with accommodation for a large number of patients, and with many clinics.

There is a large University library and a large general library (in the Landes museum "Joenaneum"), while all the clinics possess small reference libraries available for the student. The University itself is not old; it was founded in 1585, and the fine University buildings, with their attached institutes, are quite modern. Matriculation, the conditions for which are similar to those at other Austrian centres, can only be done in April; the fee is 6 kronen 20 heller. Lectures are charged at the rate of 2 kronen 10 heller per course, except where specially noted at a higher honorarium. Particulars of all lectures will be found in the official University programme, obtainable from all publishers and local booksellers, or in Vienna, for 20 heller.

Vacation courses, for practitioners only, are given during the autumn at the various clinics. These are thoroughly practical and rank among the best that are given at any Austrian centre, and they can be heartily recommended to graduates. For the last three years they have been very well attended, and early application is therefore necessary. The conditions of membership are similar to those at the Vienna vacation courses. In each case membership is limited and the fees are low.

The free-lance will find Graz an unusually pleasant centre. The amount of clinical material, owing to the concentration that exists and the fact that the hospitals tap a very large district, is very rich and varied, and there is plenty of opportunity for special and research work. This is especially the case in surgery. At Professor von Hacker's clinic there is an abundance of material, both in the in- and out-patient departments. Graz will possess a very fine new surgical clinic within the next few years which will greatly add to its clinical attractions. This new hospital is at present being built, and, judging from the plans, it promises to be one of the best on the Continent. At Von Hacker's clinic the graduate will see excellent surgery, particularly in intestinal and bone work, and will find plenty of opportunity for practical work. The chief assistants—Drs. Streissler, Finsterer, and Fischer—give regular courses on practical subjects, and, where possible, courses for practitioners. Dr. Streissler tells us that he is quite willing to give such special courses to English-speaking graduates on terms similar to those at Vienna, and as the amount of material at his disposal is very large and equally rich, such courses should prove admirably suited to the needs of surgical students. At the other clinics in connection with the Allgemenies Krankenhaus such courses can also be arranged for with the professors and assistants, and here, too, the material is rich and varied.

The other hospitals are the Landes Krankenhaus, the asylums, the large infirmary of the Barmherzige Bruder (near the Sudbahnhof Station), and the Anna Kinderspital (Franck Strasse). In addition there are several smaller hospitals. The Anna Children's Hospital is specially worth visiting. Here in the medical division the graduate will find Professor Langer, who gives a special course on diseases of children. On the surgical side is Dr. Hans Spitzy, whose pioneering work on nerve surgery has gained him a well-deserved European reputation. Dr. Spitzy, who speaks fluent English, is prepared to give practical courses on orthopædics and surgical diseases of children to English graduates. The material at his disposal is considerable and very varied, and as a teacher he is one of the best post-graduate demonstrators we have had the pleasure of listening to. Graduates who wish to join his courses will find that they leave little to be desired. The work at the Children's Hospital is very interesting from a graduate point of view, and whether he comes to Graz to join a course or only to spend a few days, the student should not miss the opportunity of visiting this excellent institution.

The points that make this centre so attractive are the concentration and the richness of its clinical material. To these must be added the fact that there is comparatively little crowding. The graduate has a chance of working quietly, of living cheaply, and of combining business with pleasure in a way which no other centre can rival. The town offers the usual diversions in the shape of theatre, numerous concerts, etc., that obtain in moderate-sized cities, and we have already alluded to the attractions which it holds out in other directions. From a clinical point of view we cannot too much emphasise the attractions of the two chief hospitals. They are thoroughly deserving of a closer acquaintance on the part of the graduate student. Comparisons we do not wish to make, but we would advise the student who comes to Austria to visit Graz before he decides to ignore it. Those who desire to study here should communicate with Docent Dr. Spitzy, Anna Kinderspital, Graz, or with Dr. E. Streissler, Chirurgische Klinik, Allegemenies Krankenhaus, Graz, who are willing to give information as to courses for graduates. In each case the needs of the applicant, as to speciality, time, etc., should be clearly and concisely stated.

